# The impact of obesity and weight loss treatment on metabolic parameters, cardiovascular autonomic and sensory nerve function and *in vitro* fertilization outcomes in infertile women: a pilot study

**DOI:** 10.3389/fendo.2025.1548587

**Published:** 2025-05-20

**Authors:** Anna Vágvölgyi, Viktor Vedelek, Nóra Keller, Dalma Szöllősi, Szilvia Lada, Attila Nemes, Péter Kempler, Adrienn Menyhárt, István Baczkó, Tamás Várkonyi, Csaba Lengyel, János Zádori

**Affiliations:** ^1^ Endocrinology and Diabetology Center, Department of Medicine, Albert Szent-Györgyi Medical School, University of Szeged, Szeged, Hungary; ^2^ Department of Genetics, Faculty of Science and Informatics, University of Szeged, Szeged, Hungary; ^3^ Institute of Clinical Pharmacy, Albert Szent-Györgyi Medical Centre, University of Szeged, Szeged, Hungary; ^4^ Institute of Reproductive Medicine, Albert Szent-Györgyi Medical Centre, University of Szeged, Szeged, Hungary; ^5^ Directorate of Nursing Management and Professional Education, Albert Szent-Györgyi Medical Centre, University of Szeged, Szeged, Hungary; ^6^ Department of Medicine and Oncology, Semmelweis University, Budapest, Hungary; ^7^ Department of Pharmacology and Pharmacotherapy, Centre of Excellence for Interdisciplinary Research, Development and Innovation, Albert Szent-Györgyi Medical School, University of Szeged, Szeged, Hungary

**Keywords:** obesity, BMI – body mass index, cardiovascular autonomic nerve function, peripheral sensory nerve function, metabolic parameters, IVF – *in vitro* fertilization

## Abstract

**Introduction:**

The global rise in obesity is linked to metabolic disorders, such as neuropathy and infertility. Our study aimed to analyze the effects of preconceptional weight loss in infertile women with obesity on peripheral sensory and cardiovascular autonomic nerve function, metabolic parameters, and the success of *in vitro* fertilization (IVF).

**Methods:**

A retrospective cohort study included women with obesity and infertility undergoing weight optimization before IVF, alongside age-matched controls. Clinical and laboratory parameters, cardiovascular autonomic and peripheral sensory function, and body composition were evaluated before and following weight loss therapy.

**Results:**

Patients with obesity [n=58; mean ± SD; age: 33.1 ± 5.42 years; body mass index (BMI): 39.3 ± 6.90 kg/m^2^] had higher resting blood pressure, prevalence of metabolic disorders, and medication usage than controls (n=45; age: 32.1 ± 7.67 years; BMI: 21.1 ± 2.02 kg/m²). Laboratory findings indicated differences in blood cell counts, glucose metabolism markers, kidney and liver functions, and lipid profile between the groups. Cardiovascular autonomic function tests indicated impairment in Valsalva-ratio (1.4 ± 0.22 vs. 1.5 ± 0.23, p<0.001) and 30/15 ratio (1.07 ± 0.12 vs. 1.12 ± 0.13, p<0.05) in patients with obesity. Peripheral sensory function tests revealed significant deterioration in vibration sense and the current perception threshold of the median nerve at 2000 Hz in patients with obesity, as compared to controls. Before and following weight loss therapy no statistically significant difference was found on cardiovascular autonomic and peripheral sensory function. Following weight loss therapy with lifestyle/dietary intervention and liraglutide, 16 female patients with obesity attended the follow-up measurement. They achieved significant weight reduction (104.3 ± 16.64 vs. 89.1 ± 15.74 kg; p<0.05) and 8 became pregnant (5 via IVF, 3 spontaneously).

**Conclusion:**

Peripheral sensory neuronal impairments were detected in infertile women with obesity compared to the controls with normal BMI. Cardiovascular autonomic dysfunction was revealed by 30/15 and Valsalva-ratio in patients with obesity, suggesting the presence of parasympathetic dysfunction. Preconceptional weight loss improved metabolic parameters. Of the infertile female patients with obesity who reached their preconceptional target weight, 18.75% achieved spontaneous pregnancy without IVF, and 62.5% of those who underwent IVF successfully conceived.

## Introduction

The worldwide prevalence of obesity has surged in recent decades, reaching pandemic levels. According to the latest data from the World Obesity Federation’s Global Obesity Observatory (1), the prevalence of overweight status and obesity among Hungarian adult women is 29.2% and 22.7%, respectively. Obesity has been recognized as the second most significant metabolic risk factor for neuropathy, following diabetes (2). In recent cross-sectional, observational studies (3, 4), a high prevalence of neuropathy was discovered in obese and normoglycemic individuals with a body mass index (BMI) exceeding 35 kg/m^2^ when compared to lean controls. Impairments in peripheral sensory neuronal and sudomotor function as well as cardiovascular autonomic dysfunction were observed in female patients with obesity compared to controls with a normal BMI (5). Although there are no specific pharmacological treatments targeting the underlying mechanisms of autonomic neuropathy, several studies indicate that newer glucose-lowering agents, such as glucagon-like peptide-1 (GLP-1) receptor agonists, may have beneficial effects. Comprehensive clinical trial data have demonstrated their role in preventing cardiovascular events (6). A study that investigated the association between extensive anthropometric measurements and neuropathy found that female sex was also significantly correlated with neuropathy (4). Obesity, as a major public health problem, contributes to a wide range of comorbidities including cardiovascular and metabolic diseases, cancers, obstructive sleep apnea, dementia, decreased quality of life and life expectancy, as well as reproductive dysfunction (7). In their recent review, Koatz and Souza found that while two studies suggested female obesity did not affect *in vitro* fertilization (IVF) outcomes, the remaining 13 indicated a negative impact on some parameters, although consensus on the effect of weight reduction was lacking (8). However, a recent study investigating 537 patients undergoing IVF with or without intracytoplasmic sperm injection (ICSI) cycles with successful oocyte retrieval could not identify a convincing correlation between endometrial thickness development, patients’ BMI, and clinical pregnancy outcome (7). Alongside these findings, obesity is a major risk factor for gestational complications (9). Based on several studies, weight loss prior to IVF procedures has been shown to significantly improve pregnancy rates and live birth rates (10–13). Furthermore, studies have reported a reduction in the number of IVF cycles required to achieve pregnancy following weight loss interventions (14). Lifestyle modification is recommended as a first-line treatment in obese, infertile women with polycystic ovarian syndrome (PCOS) (15). There is emerging evidence from randomized clinical trials that physical activity itself may improve pregnancy rates in women with reproductive health problems (16).

The aim of the present study is to evaluate the effects of preconceptional weight loss in infertile, women with obesity on peripheral sensory and cardiovascular autonomic function, metabolic and endocrine parameters, and IVF success, as well as to identify potential correlations between these factors. To our knowledge, the occurrence of cardiovascular autonomic and sensory neuropathy in this specific patient group has not been studied so far, yet it appears to be a relevant question. Additionally, it is also important to explore whether weight loss has a beneficial effect on any potential abnormalities found.

## Subjects and methods

### Study subjects

**Figure 1:**
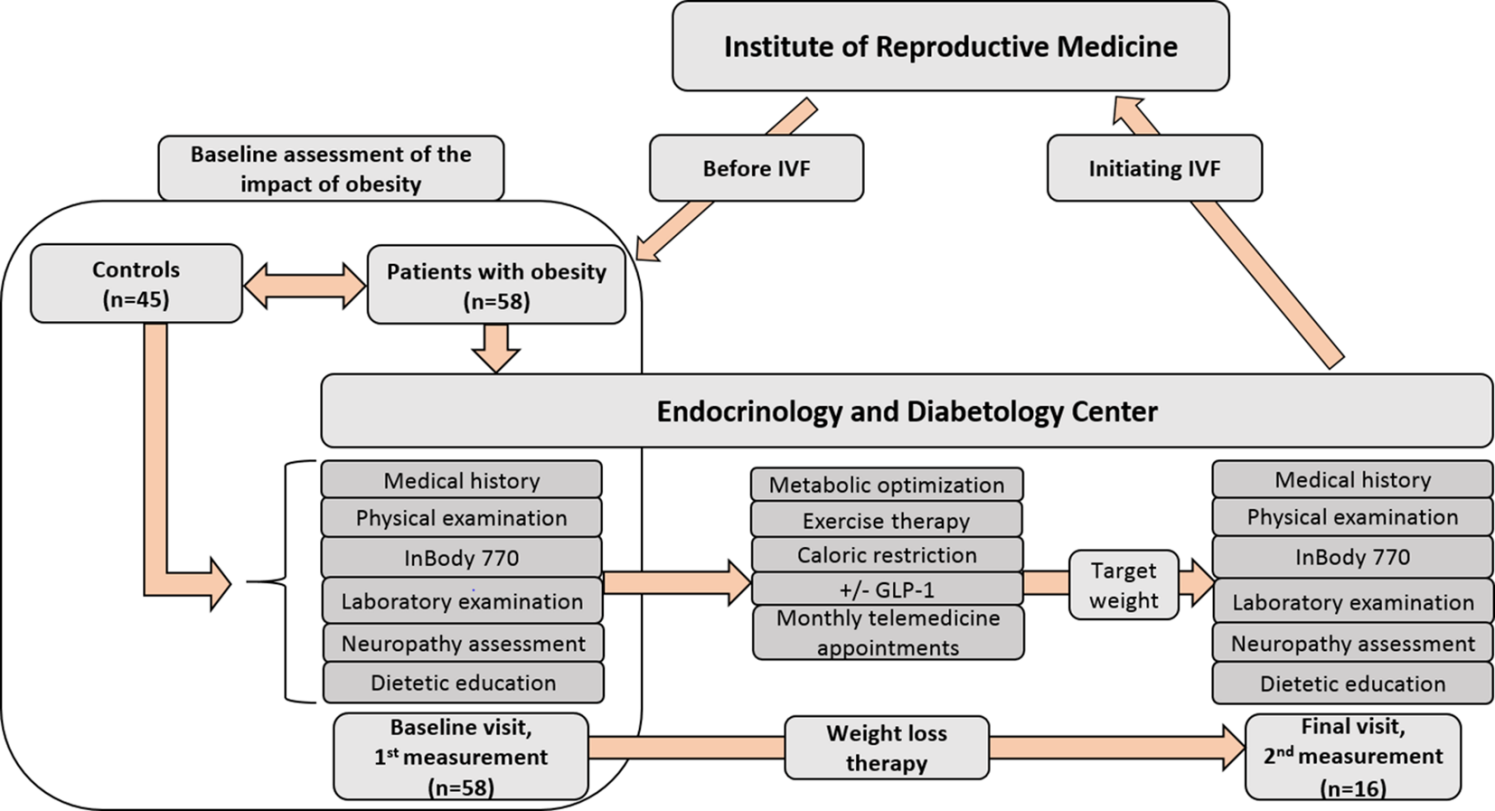
Design of the study with the number of participants. Patients with a BMI over 30 kg/m² were referred from the Institute of Reproductive Medicine to the Endocrinology and Diabetology Outpatient Clinic, University of Szeged, for endocrinologist-led preconceptional weight loss therapy. Obesity management followed the latest Hungarian guidelines (18), including the option of GLP-1 therapy. After an initial assessment—comprising laboratory tests, body composition analysis, and neuropathic evaluation—participants received personalized dietary counseling and tailored physical activity recommendations. They maintained monthly email contact with their physician and had access to immediate consultation if needed. The target weight was set collaboratively by the patient and IVF specialist, considering age and fertility status. The final visit, before IVF initiation, included a reassessment of neuropathic status, body composition, and laboratory tests as needed, along with further dietary education. *IVF, in vitro fertilization; GLP-1, glucagon like peptide analogue therapy*.

A single-center cohort study was conducted at the Endocrinology and Diabetology Outpatient Clinic of the Department of Medicine, Albert Szent-Györgyi Medical School, University of Szeged, in cooperation with the Institute of Reproductive Medicine, University of Szeged. Data collection was performed prospectively from January 1, 2020, to March 31, 2024, among women undergoing Assisted Reproductive Technology (ART) treatment, aiming for weight optimization before the planned reproductive procedure, and age-matched healthy volunteers with normal BMI (18.5-24.99 kg/m^2^) and no known history of fertility issues as controls for baseline comparison. The selection of controls for the comparison of baseline parameters was conducted using data from our QT Registry (17), which has been operating since 2019 and prospectively collects medical history, biometric data, laboratory and diagnostic findings, and results of neuropathy assessments from both healthy and non-healthy individuals. Controls were selected from the period between January 1, 2020, and March 31, 2024, with inclusion criteria ensuring a normal BMI (18.5–24.9 kg/m²), an age distribution comparable to that of the patient group, and the absence of major diseases. None of the selected controls had a known history of infertility. There were no exclusion criteria for participation in the study, apart from not meeting the inclusion criteria. All of the participants were of Caucasian origin. Our study represents a retrospective analysis of prospectively collected observational real-world data. The neuropathic investigations had been carried out in accordance with the Declaration of Helsinki (2000) of the World Medical Association and had been approved by the Hungarian Medical Research Council (approval no. 219 31891-5/2019/EÜIG), and based on this approval, it was approved by the Regional and Institutional Review Board of Human Investigations at the University of Szeged on October 21, 2019. All subjects had provided written informed consent to participate. The retrospective analysis of the effects of preconceptional weight reduction had also been conducted in accordance with the Declaration of Helsinki (2000) and had been approved by the Hungarian Medical Research Council (approval no. BM/18153-1/2023), and based on this approval, it was approved by the Regional and Institutional Review Board of Human Investigations at the University of Szeged on April 18, 2024. The treatment of obesity, including pharmacological treatment, was carried out according to recent Hungarian guidelines (18). After baseline medical assessment (laboratory tests, body composition analysis, complex neuropathic examination), participants received personalized dietary counseling from a dietitian as part of their lifestyle modification. Increasing physical activity was tailored to each participant individually. Patients with obesity contacted the attending physician by email monthly or at any time in case of complaints for immediate consultation. There was no time restriction regarding the duration of the weight loss therapy. The target weight was determined collaboratively with the patient and the IVF specialist, taking into account the patient’s age and fertility status. The weight loss therapy was led by the endocrinologist. The second and final visit was conducted after the target weight was achieved, prior to the initiation of IVF. This medical visit was conducted by an endocrinologist specialized in obesity, and also neuropathic status assessment, body composition analysis, laboratory tests as needed, and dietary education were performed again. Patients with obesity who reached the target weight before IVF and returned for the second, final examination were defined as finishers. The study design, including the number of participants, is presented and summarized in **Figure 1**. As an outcome, the occurrence or absence of clinical pregnancy was recorded. Clinical pregnancy was defined at 7 weeks of gestational age, as recommended by the International Committee for Monitoring Assisted Reproductive Technology (19).

### Methods

#### Ewing’s five standard cardiovascular reflex tests; autonomic score

Autonomic function was assessed using Ewing’s five standard cardiovascular reflex tests (20). The Ewing tests serve as the gold standard for diagnosing autonomic dysfunction; they offer non-invasive, clinically relevant, standardized, and reproducible data of autonomic function. Reflex tests were performed by measuring blood pressure and capturing continuous 6-lead electrocardiogram (ECG) signals. These signals were digitized using a multichannel data acquisition system (Cardiosys-A01 software, MDE Heidelberg GMBH, Heidelberg, Germany) at a sampling rate of 2 kHz, and the data were stored for subsequent analysis. Heart rate tests assess changes in parasympathetic function, while those based on blood pressure responses primarily indicate disturbances in sympathetic function (21). Heart rate changes were measured during deep inhalation and exhalation, in both lying and standing positions with a 30/15 ratio, and during and after a Valsalva maneuver (22). Systolic blood pressure changes were measured upon standing up from a lying position, while diastolic changes were recorded during a 3-minute handgrip. Cardiovascular reflex tests were scored separately: 0 (normal), 1 (borderline), 2 (abnormal). The overall autonomic score was calculated from the sum of each test result to characterize the severity of autonomic neuropathy. For a detailed description of the implementation of each test, we refer to our previous article (5).

#### Peripheral sensory nerve testing

##### Neurometer

The sensory function of peripheral nerves was evaluated using a Neurometer device (NM-01/CPT Neurometer, MDE Heidelberg GmbH, Heidelberg, Germany). This equipment enables non-invasive and simple testing, offering the possibility for quantitative analysis of sensory nerve function in various types of nerve fibers (23). Transcutaneous, low-voltage, sine-wave electrical stimulation was applied to determine the current perception threshold (CPT). This study examined the median and peroneal nerves. Surface electrodes with a diameter of 1 cm were placed on the distal phalanx of the index finger and the hallux. These electrodes were attached to intact skin surfaces to prevent peripheral sensory disturbance caused by scars and wounds. The applied stimuli had an amplitude range of 0.01 to 9.99 mA. Initially, the current intensity was gradually increased until the subject indicated sensation. Then, short stimuli lasting 2 to 5 seconds were applied at progressively lower intensities until the minimal intensity of consistent sensation was reached. CPT intensities were assessed at three different stimulation frequencies (2000 Hz, 250 Hz, 5 Hz) for both the upper and lower limbs, evaluating the function of large myelinated, small myelinated, and small unmyelinated sensory fibers.

##### 128-Hz Rydel-Seiffer graduated tuning fork

The 128-Hz Rydel-Seiffer graduated tuning fork was employed to assess the sense of vibration at the distal end of the radius and at the level of the hallux. Results of the tuning fork examination were compared to age-dependent normal values published by Martina et al. in 1998 (24). On a scale of 1-8, the normal range was 7-8, borderline was 6, and abnormal was 1-5, indicating an impaired sense of vibration.

#### Body composition analysis

The body composition analysis was performed by a dietitian with a bioelectrical impedance analysis using a segmental body composition analyzer device (InBody 770, InBodyUSA, Cerritos, CA). The following parameters were determined and documented: skeletal muscle mass (SMM, kg), free fat mass index (calculated by dividing the fat-free mass by the square of the height in meters, FFMI), body fat percentage (the total weight of fat in the body divided by the total body weight and then multiplied by 100, PBF, %), whole body phase angle (represents the relationship between resistance (R) and reactance (Xc) of the electrical current passing through the body, WBPA=arctan{Xc/R},°), bone mineral content (BMC, kg), visceral fat area (represents the amount of visceral fat in the abdominal region of the body, VFA, cm2), basal metabolic rate (BMR, kcal), and InBody score (ranging from 0 to 100, with higher scores generally indicating better body composition and overall health, IBS).

#### Laboratory data

The laboratory results obtained within one month of the appointment date at the obesity clinic were considered valid. The following parameters were collected and documented in case they were available: white blood cell count (G/L), red blood cell count (T/L), hemoglobin (g/L), hematocrit (%), mean cell volume of red blood cells (MCV, fL), platelet count (G/L), sodium (mmol/L), potassium (mmol/L), adjusted calcium (mmol/L), magnesium (mmol/L), glucose (mmol/L), hemoglobin A1c% (HbA1c; %), insulin (mIU/L), estimated glomerular filtration rate (eGFR; ml/min/1.73 m²), uric acid (μmol/L), total protein (g/L), albumin (g/L), total cholesterol (mmol/L), triglyceride (mmol/L), HDL-cholesterol (mmol/L), LDL-cholesterol (mmol/L), aspartate aminotransferase (ASAT/GOT; U/L), alanine aminotransferase (ALAT/GPT; U/L), γ-glutamyl transferase (GGT; U/L), total bilirubin (μmol/L), direct/conjugated bilirubin (μmol/L), alkaline phosphatase (U/L), amylase (U/L), lipase (U/L), C-reactive protein (CRP; mg/L), ferritin (ng/mL), iron (μmol/L), thyroid-stimulating hormone (TSH; mIU/L), free triiodothyronine (fT3; pmol/L), free thyroxine (fT4; pmol/L), anti-thyroglobulin (anti-TG; IU/mL), anti-thyroid peroxidase (anti-TPO; IU/mL), anti-Müllerian hormone (AMH; ng/ml), testosterone (nmol/L), sex hormone binding globulin (SHBG; nmol/L), dehydroepiandrosterone sulfate (DHEAS; µmol/L), cortisol (nmol/L), prolactin (mIU/L), parathormone (pmol/L), 25-hydroxy-D3- and D2-vitamin (25OHD3/D2-vitamin; nmol/L). The following urinary parameters were collected and documented: total protein (mg/dL), albumin (mg/L), urine albumin/creatinine ratio (ACR; mg/mmol), urine nitrite, urine pH, urine protein, urine glucose, urine ketone body, urine urobilinogen, urine bilirubin, urine white blood cell, urine red blood cell. Homeostatic model assessment for insulin resistance (HOMA-IR index) was calculated using the formula: fasting glucose (mmol/L) × fasting insulin (mIU/L)/22.5.

#### Weight loss therapy

##### Pharmacological therapy

Patients with a BMI over 30 kg/m² were referred from the Institute of Reproductive Medicine to the Endocrinology and Diabetology Outpatient Clinic at the University of Szeged for preconception weight loss therapy supervised by an endocrinologist (**Figure 1**). The therapeutic approach to obesity, encompassing dietary, lifestyle and pharmacological components, adhered to the most recent Hungarian clinical guidelines (18), including the option of GLP-1 receptor agonist therapy. During the study period, liraglutide was the only GLP-1 receptor agonist available in Hungary for the treatment of obesity. Due to its documented cardiovascular benefits (25, 26), and the advantage of a short washout period prior to IVF cycle initiation, liraglutide was administered to a substantial proportion of patients who consented to pharmacological therapy. In this study population, neither orlistat nor the fixed-dose combination of naltrexone and bupropion was utilized. Pharmacological treatment was not mandatory; rather, therapy selection was based on a shared decision-making process, with a patient-tailored approach to identify the most feasible treatment option for each individual. It is important to note that preconception endocrinological care addressed not only obesity but also the identification and optimization of all other endocrine and metabolic disorders, as managed by the endocrinologist.

##### Physical activity

Following an initial assessment—which involved laboratory testing, body composition analysis, and neuropathic evaluation—participants received individualized dietary counseling by a dietitian and personalized physical activity recommendations by both the physician and the dietitian. The goal was to reach at least the minimum physical activity level recommended by the World Health Organization, which is either 150 minutes of moderate-intensity physical activity per week, 75 minutes of vigorous-intensity physical activity per week, or a combination of both (27). Based on the patient actual lifestyle and the general fitness level, individualized recommendations were provided to gradually increase daily physical activity. These recommendations included suggestions for incorporating suitable forms of exercise, considering the patient’s possibilities, along with guidance on duration and frequency. Patients were encouraged to monitor their daily physical activity by combining cardio-type exercise with resistance training, supported by the use of freely available online applications. Weekly weight measurements were recommended, along with monthly email check-ins with the treating physician.

##### Dietary intervention

The dietary intervention for patients with obesity was based on the national professional guideline on adult obesity management (18), and the Nutrition Care Process (28). An individualized energy deficit of approximately 500–1000 kcal/day was applied while ensuring the prevention of nutrient deficiencies. To help sustain the individualized energy deficit determined by InBody analysis, patients were encouraged to engage in calorie tracking. For this purpose, they were advised to use freely accessible mobile applications throughout the duration of the weight loss therapy. The structure and timing of meals also played a key role; meal plans typically consisted of three main meals and two snacks per day, consistent with national nutritional guidelines (29), and were individually adjusted and reviewed as needed. Macronutrient distribution adhered to these recommendations, with daily energy intake composed of 45–60% carbohydrates, 15–20% protein, and 25–35% fat. Carbohydrates were primarily derived from low glycemic index sources, and fats mainly from unsaturated fatty acids. In case of any dietary questions, follow-up consultations with the dietitians were also available. Prior to IVF procedures, a final dietetic consultation was conducted to review nutritional progress and reinforce individual goals.

#### Statistical analyses

In this study, we retrospectively analyzed the prospectively collected data. Statistical significance was determined using Welsch’s two-tailed significance test. Cohen’s effect size (d) was determined for each dimension and statistical power was calculated. Correlation was determined using Pearson coefficient and the corresponding two-sided p-values were also calculated. All of the analyses including Kaplan Meier survival curve were implemented in Python 3.6 using statsmodels, NumPy, SciPy, Pandas, and Lifelines libraries. The primary power calculation analyses focused on the decrease of BMI, we estimated a mean value of BMI of the obese group of 33.8 ± 3.3 and expected a 10% decrease in the mean BMI with p<0.05 and power of 80%. Based on this calculation a total number of 30 measurements 15 baseline measurements and 15 2^nd^ measurements would be sufficient, to determine if the weight loss is significant. Additionally, due to the low number of participants who returned for the second measurement (n=16), we conducted post-hoc power calculations and estimated the required sample sizes to achieve at least p<0.05 significance, considering Cohen’s effect size (d) for each individual dimension. This provides a reliable estimate of the trustworthiness of the calculated significance individually for every measured data. If the sample size is close to or smaller than the number of measurements, the chance of a significant difference is high. Additionally, for critical assessment of the sample sizes and p-values we provided *post-hoc* power % values for significant changes based on https://clincalc.com/stats/Power.aspx. We conducted the calculations using an alpha level of 0.05. Details regarding significance, Cohen’s effect size, estimated sample sizes and power % are available in the **Supplementary Table**.

## Results

### Baseline clinical data in infertile women with obesity and in controls

In our study we included 58 infertile female patients with obesity (mean ± SD; age: 33.1 ± 5.42 years; BMI: 39.3 ± 6.90 kg/m^2^) prior to undergoing lifestyle changes aimed at achieving weight loss, with or without medical treatment. Additionally, 45 age-matched female volunteers with a normal BMI (age: 32.1 ± 7.67 years; BMI: 21.1 ± 2.02 kg/m^2^) were enrolled as controls. The relevant baseline clinical data of the subjects are shown in **Table 1**.

**Table 1:** Relevant clinical data in the two study groups at baseline.

Clinical Data	Controls (n=45)	Infertile Patients with Obesity (n=58)	P-value
Age (year)	32.1 ± 7.67	33.1 ± 5.42	n.s.
Height (cm)	168.0 ± 6.16	166.7 ± 6.19	n.s.
Weight (kg)	59.6 ± 6.20	109.5 ± 21.59	<0.001
Body Mass Index (kg/m^2^)	21.1 ± 2.02	39.3 ± 6.90	<0.001
Waist circumference (cm)	69.0 ± 5.46	108.0 ± 15.12	<0.001
Hip circumference (cm)	94.7 ± 5.09	127.1 ± 15.94	<0.001
Waist-to-hip ratio	0.73 ± 0.05	0.86 ± 0.13	<0.001
Systolic BP (mmHg)	113.4 ± 13.87	136.8 ± 14.55	<0.001
Diastolic BP (mmHg)	69.4 ± 11.48	84.7 ± 11.98	<0.001
Smoking history	7 (16%)	18 (31%)	n.s.
Alcohol consumption	10 (22%)	9 (16%)	n.s.
Impaired glucose tolerance	0 (0%)	16 (28%)	<0.001
Type 2 diabetes mellitus	0 (0%)	5 (9%)	<0.05
Type 1 diabetes mellitus	0 (0%)	1 (2%)	n.s.
Hypertension	0 (0%)	17 (29%)	<0.001
Polycystic ovarian syndrome	5 (11%)	32 (55%)	<0.001
Hirsutism	1 (2%)	32 (55%)	<0.001
Hypothyroidism	0 (0%)	17 (29%)	<0.001
Endometriosis	2 (4%)	3 (5%)	n.s.
Medication			
Metformin	2 (4%)	41 (71%)	<0.001
β-blocker	0 (0%)	8 (14%)	<0.01
ACE inhibitor or ARB	1 (2%)	3 (5%)	n.s.
Ca-antagonist	0 (0%)	2 (3%)	n.s.
Imidazolidine receptor agonist	0 (0%)	1 (2%)	n.s.
α_2_ adrenergic receptor agonist	0 (0%)	9 (16%)	<0.01
Statin	1 (2%)	0 (0%)	n.s.
ASA	0 (0%)	4 (7%)	<0.05
Diuretics	0 (0%)	1 (2%)	n.s.
Dopamine agonist	1 (2%)	2 (3%)	n.s.
Levothyroxine	0 (0%)	13 (22%)	<0.001

BP, blood pressure; ACE, angiotensin-converting enzyme; ARB, angiotensin-receptor blocker; Ca, calcium; ASA, acetylsalicylic acid; PAI, platelet aggregation inhibitor.n.s., non-significant.

In terms of age, height, prior pregnancies, miscarriages, abortions, previous unsuccessful IVF attempts, number of children, cesarean sections, history of endometriosis, smoking, and alcohol consumption, there were no differences between the group of infertile women with obesity and the control group.

The patients with obesity had significantly higher resting mean systolic (113.4 ± 13.87 vs. 136.8 ± 14.55 mmHg; p<0.001) and diastolic blood pressure (69.4 ± 11.48 vs. 84.7 ± 11.98 mmHg, p<0.001) than the controls. Infertile patients with obesity experienced significantly higher rates of hypertension (0 vs. 17, p<0.001), impaired glucose tolerance (0 vs. 16; p<0.001), type 2 diabetes (0 vs. 5; p<0.05), polycystic ovarian syndrome (5 vs. 32; p<0.001), hirsutism (1 vs. 32; p<0.001), and hypothyroidism (0 vs. 17; p<0.001).

Significantly more patients with obesity in the infertile group took metformin, β-blocker, α_2_ adrenergic receptor agonist, acetylsalicylic acid, and levothyroxine but there was no significant difference between the two groups with respect to the usage of angiotensin-converting enzyme inhibitors, or angiotensin receptor blockers, Ca^2+^-channel blockers, imidazolidine or α_2_ adrenergic receptor agonists, statins, and diuretics (**Table 1**).

### Laboratory data of infertile women with obesity and control subjects

The relevant clinical data for patients with obesity and controls are displayed in **Table 2**. White and red blood cell count, thrombocyte, sodium, glucose, insulin, HbA1c, uric acid, triglyceride, LDL-cholesterol, ASAT/GOT, ALAT/GPT, GGT, alkaline phosphatase, ferritin and CRP values were significantly higher in patients with obesity compared to controls with a normal BMI. The mean cellular volume, serum phosphate, albumin, creatinine, HDL-cholesterol, amylase, lipase, iron, and SHBG levels were significantly lower among patients with obesity. There were no significant differences in other laboratory parameters between the two groups. Due to the individualized nature of ambulatory patient care, not all patients with obesity and controls had all the collected investigated parameters determined. Given that blood samples from patients with obesity were not always collected on days 3–5 of the menstrual cycle, the objective comparison of FSH, LH, and estradiol hormone levels was not possible due to their cycle sensitivity.

**Table 2:** Relevant laboratory data in the two study groups at baseline.

Laboratory Data	Controls	Infertile Patients with Obesity	P-value
White blood cell count (G/L)	6.3± 1.36 (n=44)	8.4 ± 2.02 (n=54)	<0.001
Red blood cell count (T/L)	4.5 ± 0.28 (n=44)	4.8 ± 0.38 (n=55)	<0.001
Hemoglobin (g/L)	133.0 ± 9.22 (n=44)	134.2 ± 19.11 (n=55)	n.s.
Hematocrit (L/L)	0.39 ± 0.02 (n=44)	0.41 ± 0.03 (n=55)	n.s.
Mean cellular volume (fL)	87.2 ± 3.50 (n=44)	84.1 ± 8.56 (n=55)	<0.05
Thrombocyte (G/L)	256.1 ± 59.93 (n=44)	318.4 ± 62.46 (n=55)	<0.001
Sodium (mmol/L)	138.2 ± 2.05 (n=43)	139.2 ± 2.37 (n=55)	<0.05
Potassium (mmol/L)	4.3 ± 0.34 (n=43)	4.6 ± 1.46 (n=55)	n.s.
Adjusted calcium (mmol/L)	2.3 ± 0.06 (n=40)	2.3 ± 0.09 (n=50)	n.s.
Magnesium (mmol/L)	0.9 ± 0.06 (n=39)	0.9 ± 0.07 (n=49)	n.s.
Glucose (mmol/L)	4.7 ± 0.50 (n=45)	5.3 ± 0.98 (n=58)	<0.001
Insulin (mIU/L)	5.8 ± 4.49 (n=21)	16.3 ± 19.67 (n=42)	<0.01
HOMA-IR	1.3 ± 1.00 (n=21)	4.0 ± 4.96 (n=40)	<0.05
HbA1c (%)	5.2 ± 0.26 (n=40)	5.5 ± 0.67 (n=55)	<0.01
eGFR (mL/min/1.73m^2^)	91.1 ± 12.91 (n=43)	95.3 ± 14.20 (n=53)	n.s.
Uric acid (μmol/L)	219.1 ± 45.79 (n=40)	328.59 ± 71.46 (n=50)	<0.001
Total protein (g/L)	73.4 ± 3.80 (n=42)	70.9 ± 11.22 (n=45)	n.s.
Albumin (g/L)	48.2 ± 2.13 (n=43)	46.7 ± 2.66 (n=49)	<0.01
Total cholesterol (mmol/L)	4.9 ± 0.87 (n=42)	4.9 ± 0.74 (n=51)	n.s.
Triglyceride (mmol/L)	0.9 ± 0.37 (n=42)	1.6 ± 0.86 (n=51)	<0.001
HDL-cholesterol (mmol/L)	1.8 ± 0.42 (n=37)	1.2 ± 0.28 (n=51)	<0.001
LDL-cholesterol (mmol/L)	2.5 ± 0.74 (n=36)	2.9 ± 0.64 (n=50)	<0.01
ASAT/GOT (U/L)	19.0 ± 6.54 (n=45)	24.4 ± 18.27 (n=53)	<0.05
ALAT/GPT (U/L)	18.1 ± 8.78 (n=45)	30.4 ± 16.53 (n=53)	<0.001
Gamma GT (U/L)	11.5 ± 4.77 (n=45)	27.9 ± 22.19 (n=52)	<0.001
Total bilirubin (μmol/L)	9.6 ± 5.91 (n=45)	8.0 ± 3.51 (n=50)	n.s.
Alkaline phosphatase (U/L)	56.2 ± 13.51 (n=45)	77.2 ± 22.08 (n=50)	<0.001
C-reactive protein (mg/L)	1.5 ± 1.06 (n=39)	9.1 ± 8.34 (n=42)	<0.001
Ferritin (ng/mL)	38.4 ± 21.62 (n=17)	76.71 ± 56.45 (n=38)	<0.001
Iron (μmol/L)	16.8 ± 7.94 (n=39)	13.6 ± 5.62 (n=50)	<0.05
TSH (mIU/L)	2.6 ± 1.52 (n=42)	2.1 ± 1.32 (n=50)	n.s.
fT3 (pmol/L)	5.1 ± 0.79 (n=17)	4.9 ± 0.73 (n=15)	n.s.
fT4 (pmol/L)	16.2 ± 2.12 (n=19)	17.1 ± 5.00 (n=18)	n.s.
Anti-TG (IU/mL)	49.8 ± 77.37 (n=19)	27.0 ± 58.17 (n=40)	n.s.
Anti-TPO (IU/mL)	19.0 ± 40.45 (n=17)	39.8 ± 75.69 (n=42)	n.s.
Parathormone (pmol/L)	3.2 ± 1.36 (n=34)	4.7 ± 6.13 (n=48)	n.s.
25OHD3/D2-vitamin (nmol/L)	81.0 ± 42.04 (n=36)	65.8 ± 22.47 (n=47)	n.s.
Testosterone (nmol/L)	1.1 ± 0.65 (n=24)	1.3 ± 0.74 (n=44)	n.s.
SHBG (nmol/L)	77.0 ± 40.34 (n=25)	33.7 ± 24.33 (n=44)	<0.001
DHEAS (umol/L)	6.2 ± 3.18 (n=22)	7.1 ± 3.11 (n=42)	n.s.
Cortisol (nmol/L)	345.9 ± 102.33 (n=17)	354.8 ± 149.95 (n=34)	n.s.
Prolactin (mIU/L)	370.6 ± 190.31 (n=23)	401.4 ± 229.51 (n=43)	n.s.

The data are presented as mean ± SD. HOMA-IR, homeostasis model assessment of insulin resistance; HbA1c, hemoglobin A1c; eGFR, estimated glomerular filtration rate; ASAT/GOT, aspartate aminotransferase; ALAT/GPT, alanine aminotransferase; GGT, γ-glutamyl transferase; TSH, thyroid-stimulating hormone; fT3, free triiodothyronine; fT4, free thyroxine; Anti-TG, anti-thyroglobulin; Anti-TPO, anti-thyroid peroxidase; 25OHD3/D2-vitamin, 25-hydroxy-D3- and D2-vitamin; SHBG, sex hormone binding globulin; DHEAS, dehydroepiandrosterone sulfate.n.s., non-significant.

The baseline AMH value for the infertile female patients with obesity (n=36) was 3.6 ± 3.80 ng/ml. Regrettably, only 2 of the finishers had their AMH values measured at baseline, with a mean value of 10.0 ± 6.91 ng/ml.

### Cardiovascular autonomic function tests of infertile women with obesity and control subjects

A significant impairment in Valsalva-ratio (1.5 ± 0.23 vs 1.4 ± 0.22; p<0.001) and also in 30/15 ratio (1.12 ± 0.13 vs. 1.07 ± 0.12; p<0.05) in patients with obesity was detected compared to controls. No further significant differences in the results of autonomic tests could be detected between the two groups. The autonomic score (1.2 ± 1.33 vs. 2.3 ± 1.83; p<0.001), characterizing the cardiovascular autonomic neuropathy based on the summary of the results of the Ewing tests, was significantly higher among patients with obesity compared to the controls.

### Peripheral sensory function in infertile female patients with obesity and control subjects

The 128-Hz Rydel-Seiffer graduated tuning fork test (**Table 3**) revealed an impaired sense of vibration symmetrically on all four limbs at the level of radius and halluces in the infertile patients with obesity compared to controls.

**Table 3:** Peripheral sensory function testing in the study groups by 128-Hz Rydel-Seiffer graduated tuning fork on the distal end of right (RR) and left (LR) radius and the right (RH) and left (LH) hallux in infertile women with obesity and in controls.

Rydel-Seiffer graduated tuning fork tests	Controls (n=45)	Infertile Patients with Obesity (n=57)	P-value
RR	7.71 ± 0.46	7.37 ± 0.58	<0.01
LR	7.62 ± 0.49	7.30 ± 0.72	<0.01
RH	7.58 ± 0.54	6.97 ± 0.89	<0.001
LH	7.47 ± 0.63	6.98 ± 0.74	<0.001

RR, right radius, LR, left radius, RH, right hallux, LH, left hallux.

A significantly elevated CPT was observed in patients with obesity at the median nerve at the tested frequency of 2000 Hz (172.133 ± 39.9 vs. 198.365 ± 62.183; p<0.05) using the Neurometer. At rest, tested frequencies at the median and the peroneal nerve did not show a statistically significant discrepancy in the CPT values.

### Body composition analysis

All InBody parameters (**Table 4**) exhibited significant differences between the two groups, consistent with our initial expectations.

**Table 4:** Body composition analysis with InBody at baseline in infertile women with obesity and in controls.

InBody Parameters	Controls	Infertile Patients with Obesity	P-value
SMM (kg)	24.4 ± 2.53 (n=45)	32.2 ± 4.98 (n=42)	<0.001
FFMI (kg/m^2^)	15.8 ± 1.16 (n=43)	20.5 ± 2.43 (n=42)	<0.001
PBF (%)	24.7 ± 5.16 (n=45)	46.667 ± 5.19 (n=43)	<0.001
WBPA (°)	5.2 ± 0.61 (n=40)	5.6 ± 0.44 (n=41)	<0.01
BMC (kg)	2.658 ± 0.275 (n=45)	3.2 ± 0.48 (n=43)	<0.001
BMR (kcal)	1332.0 ± 91.24 (n=45)	1606.3 ± 186.21 (n=43)	<0.001
VFA (cm^2^)	63.2 ± 18.54 (n=42)	216.9 ± 59.28 (n=43)	<0.001
IBS	75.6 ± 3.96 (n=45)	57.2 ± 9.21 (n=43)	<0.001

SMM, skeletal muscle mass; FFMI, free fat muscle index; PBF (%), percent body fat; WBPA, whole body phase angle; BMC, bone mineral content; VFA, visceral fat area; BMR, basal metabolic rate; IBS, in body score.

### Results at baseline (1^st^ measurement) and after weight loss therapy (2^nd^ measurement) in infertile women with obesity; clinical pregnancy outcome

**Figure 2:**
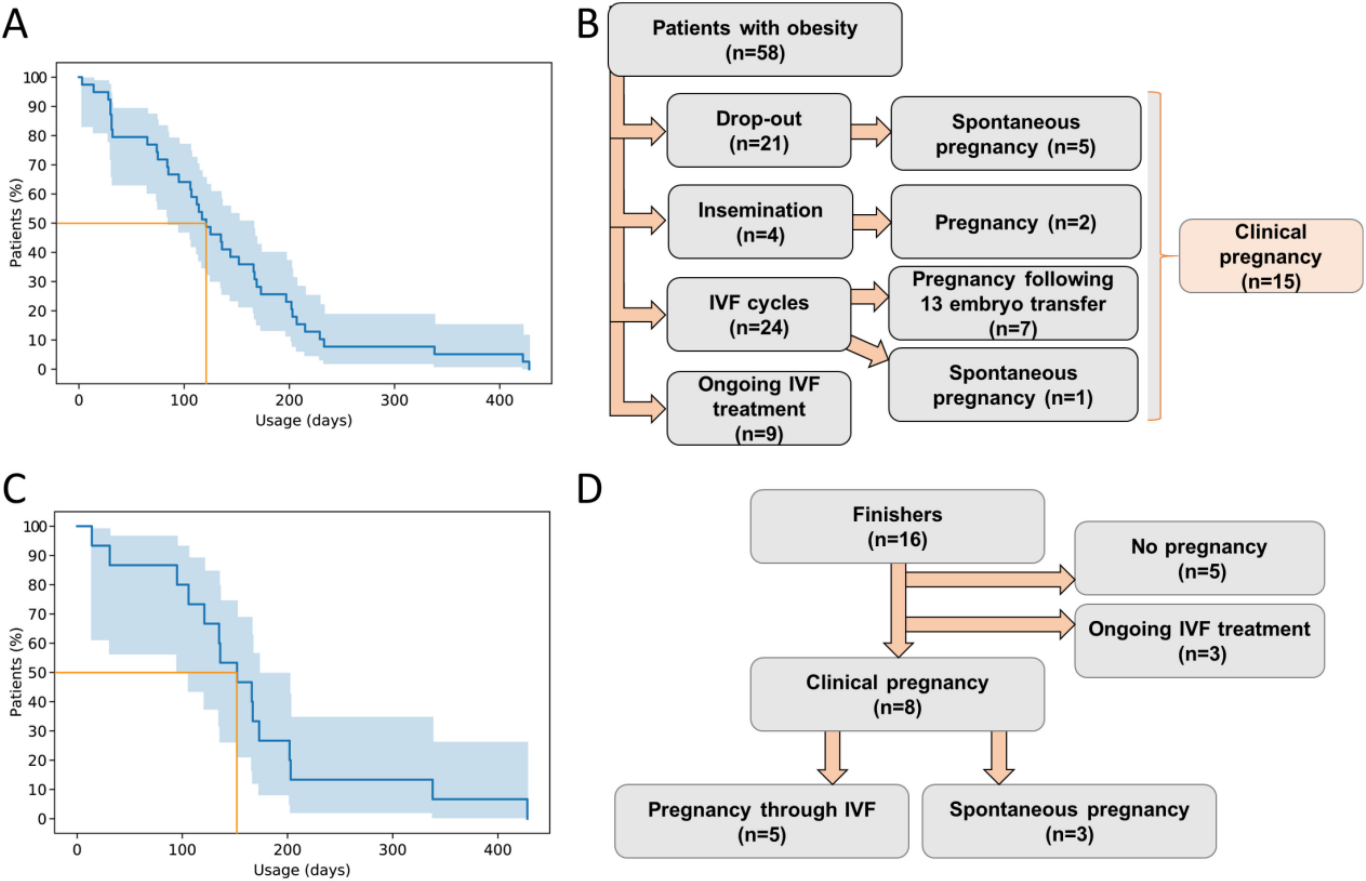
**(A)** Decrease in the number of patients continuing liraglutide treatment over time, starting with an initial sample size of 41. Blue line represents Kaplan-Meier estimator; the light blue shading represents 95% confidence interval. Orange line represents where 50% percent of the patients stop using the substance. **(B)** Flow chart represents the pregnancy outcome of 58 infertile women with obesity. **(C)** The decrease in the number of patients continuing liraglutide treatment over time, starting with the finisher sample size of 16. Blue line represents Kaplan-Meier estimator; the light blue shading represents 95% confidence interval. Orange line represents where 50% percent of the patients stop using the substance. **(D)** Flowchart represents the pregnancy outcome of the 16 finishers. *IVF, in vitro fertilization*.

In addition to lifestyle and dietary changes, 48 out of the starting 58 patients with obesity also started liraglutide therapy. Liraglutide was applied in these 48 patients with obesity for an average of 156.3 ± 129.9 days (n=42) with a mean liraglutide dose of 2.04 ± 0.57 mg (n=43). **Figure 2A** demonstrates the decrease in the number of patients with obesity continuing liraglutide treatment over time, starting with an initial sample size of 41. Out of 58 obese, infertile women presented at baseline before weight-loss therapy, 15 became pregnant, as detailed in **Figure 2B**. Out of the 58 patients with obesity, 21 dropped out (including 5 spontaneous pregnancies), 9 are still undergoing IVF treatment, 4 underwent only insemination (resulting in 2 pregnancies), 24 started IVF cycles (with 1 spontaneous pregnancy after a failed IVF), and 13 embryo transfers were performed (resulting in 7 pregnancies). Out of the 58 women, 10 did not use liraglutide. Out of the 15 pregnancies, only 1 woman became pregnant without using liraglutide for weight loss before fertility treatments; all the other pregnancies occurred in women who were pre-treated with liraglutide.

Out of the initial 58 patients with obesity, only 16 (referred to as “finishers”) attended the final measurements after achieving the target weight reduction prior to IVF, resulting in a retention rate of 27.6% within this highly motivated cohort. Consequently, the 2^nd^ measurement was conducted on these 16 patients with obesity, during which medical visit by an endocrinologist specialized in obesity, neuropathic status assessment, body composition analysis, laboratory tests as needed, and dietary education were performed again. **Figure 2C** demonstrates the decrease in the number of patients with obesity continuing liraglutide treatment over time, starting with an initial sample size of 16 of the finishers. All finishers received liraglutide therapy in addition to lifestyle/dietary changes. Liraglutide was applied for an average of 159.7 ± 103.4 days (n=16). The average liraglutide dose was 1.99 ± 0.52 mg (n=16). Among the finishers, 8 became pregnant (5 through IVF and 3 spontaneously), 3 are still undergoing IVF treatment, while 5 either did not conceive or did not continue IVF therapy. Out of 16 patients with obesity, 3 became spontaneously pregnant, meaning IVF was not required for 18.75% of the patients. Excluding these 3 patients, 5 out of the remaining 13 candidates did not ultimately continue IVF therapy. Out of the remaining 8 patients, 5 became pregnant through IVF, which represents a 62.5% success rate. The flowchart summarizing the clinical pregnancy outcomes of the 16 finishers is presented in **Figure 2D**.

The effectiveness of obesity therapy in the finishers is evidenced by the significant decrease in body weight (104.3 ± 16.64 vs. 89.1 ± 15.74 kg; p<0.05) and BMI (38.5 ± 5.02 vs. 32.9 ± 5.20 kg/m^2^; p<0.01). The average duration of treatment was 232.9 ± 170.86 days, with the starting weight of 104.3 ± 16.11 kg and the final weight of 89.09 ± 15.24 kg with an average weight loss of 15.2 ± 6.96 kg. The finishers were able to lose an average of 14.6 ± 6.06% of their initial body weight. Significant changes following the weight-loss therapy were observed only in the summarized parameters presented in **Table 5**. However, the results of cardiovascular autonomic and peripheral sensory measurements showed no statistically significant differences before and after weight loss therapy.

**Table 5:** Clinical, laboratory, and test data showing significant change from the baseline (1^st^ measurement) to after weight loss therapy (2^nd^ measurement) in infertile women with obesity.

Clinical and Laboratory Data	Patients at 1^st^ Measurement	Patients at 2^nd^ Measurement	P-value
Weight (kg)	104.3 ± 16.64 (n=16)	89.1 ± 15.74 (n=16)	<0.05
Body Mass Index (kg/m^2^)	38.5 ± 5.02 (n=16)	32.9 ± 5.20 (n=16)	<0.01
Waist circumference (cm)	108.7 ± 20.05 (n=15)	94.0 ± 13.58 (n=16)	<0.05
Hip circumference (cm)	120.1 ± 15.21 (n=15)	115.6 ± 12.54 (n=16)	n.s.
Waist-to-hip ratio	0.92 ± 0.21 (n=15)	0.81 ± 0.08 (n=15)	n.s.
Systolic BP (mmHg)	133.3 ± 12.91 (n=16)	127.5 ± 15.76 (n=16)	n.s.
Diastolic BP (mmHg)	85.0 ± 10.82 (n=16)	80.44 ± 10.46 (n=16)	n.s.
HbA1c (%)	5.4 ± 0.38 (n=15)	5.1 ± 0.18 (n=11)	<0.05
25OHD3/D2-vitamin (nmol/L)	66.9 ± 24.55 (n=15)	103.9 ± 44.09 (n=11)	<0.05
PBF%	47.0 ± 3.81 (n=13)	42.5 ± 6.50 (n=13)	<0.05

During the weight-loss therapy, the following changes were made to antihypertensive and thyroid hormone replacement medications between the 1^st^ and the 2^nd^ measurements. Although there was no significant difference in TSH levels between the 1^st^ and 2^nd^ measurements (1^st^ vs. 2^nd^ measurement; 2.2 ± 1.51 vs. 2.4 ± 1.30; n.s.) for the 16 patients with obesity who were retested, considering the latest ESHRE guideline (30) recommended TSH target levels in relation to the planned IVF, levothyroxine therapy was initiated in three cases, the dosage was increased in two further cases, and thyroid hormone replacement therapy was discontinued in one case. New antihypertensive therapy was initiated and adjusted in two cases and prenatal multivitamins were started in four cases. Based on baseline laboratory tests indicating low 25OHD3/D2 vitamin levels, vitamin D3 loading and subsequent supplementation were initiated in 7 out of 16 patients with obesity. Before starting the weight loss therapy, 11 out of the 16 patients were already on metformin treatment for various indications (polycystic ovary syndrome, impaired fasting glucose, impaired glucose tolerance, type 2 diabetes mellitus). Following the medical assessment, metformin was initiated in an additional 2 patients, so only 3 patients did not receive metformin alongside liraglutide therapy. No further pharmacological intervention was initiated by the endocrinologist overseeing the weight loss therapy.

Examining the pregnant/non-pregnant subgroups of the whole starting obese, infertile population revealed no relevant significant differences among the investigated groups.

### Correlations in the whole population

The initial body weight positively correlated with the presence of insulin resistance (r=0.550, p<0.001), IGT (r=0.299, p=0.002), hypertension (r=0.433, p<0.001), PCOS (r=0.377, p<0.001), hirsutism (r=0.404, p<0.001), and hypothyroidism (r=0.312, p=0.001).

The initial body weight also positively correlated with fasting glucose values (R=0.269, p=0.006), fasting insulin (r=0.381, p=0.002), correspondingly with the HOMA-IR index (r=0.380, p=0.003), as well as with HbA1c% (r=0.250, p=0.015), triglyceride levels (r=0.393, p<0.001), inflammatory parameters such as CRP (r=0.527, p<0.001) and ferritin (r=0.336, p=0.012), blood count elements such as white blood cell count (r=0.503, p<0.001), red blood cell count (r=0.460, p<0.001), and platelet count (r=0.322, p=0.001), liver enzymes (GOT: r=0.245, p=0.015; GPT: r=0.479, p<0.001; alkaline phosphatase: r=0.451, p<0.001; GGT: r=0.463, p<0.001), and uric acid levels (r=0.659, p<0.001), while negatively correlated with AMH values (r=-0.363, p=0.030), HDL (r=-0.534, p<0.001), amylase (r=-0,496, p<0.001), iron levels (r=-0.240567268, p=0.023), and 25OHD3/D2 vitamin levels (r=-0.270, p=0.014).

The initial body weight positively correlated with resting systolic (r=0.629, p<0.001) and diastolic (r=0.527, p<0.001) blood pressure. Regarding the results of neuropathic tests, the initial body weight positively correlated with the autonomic score (r=0.328, p<0.001), and negatively with the Valsalva ratio and 30/15 ratio, as well as with all tuning fork tests (RR: r=-0.280, p=0.004; RH: r=-0.331, p<0.001; LR: r=-0.217, p=0.029; LH: r=-0.348, p<0.001). As expected, BMI demonstrated similarly significant correlations as the initial body weight.

The autonomic score correlated negatively with three of the four tuning fork tests (RR: r = -0.200, p = 0.045; LR: r = -0.277, p = 0.005; LH: r = -0.231, p = 0.020), with the InBody score (r = -0.262, p = 0.014), and positively with the basal metabolic rate (r = 0.357, p < 0.001), with the FFMI (r = 0.320, p = 0.003), as well as with the PBF% (r = 0.273, p = 0.010).

## Discussion

### Baseline clinical data in infertile women with obesity and in controls

In the present study, infertile female patients with obesity before undergoing lifestyle changes for the purpose of weight loss had significantly higher resting mean systolic and diastolic blood pressure (**Table 1**). Consistent with the literature, infertile patients with obesity suffered significantly more often from hypertension (31), impaired glucose tolerance (32), type 2 diabetes (33), polycystic ovarian syndrome (34), hirsutism (35), and hypothyroidism (36) than the controls. Significantly more patients with obesity took metformin, β-blocker, α_2_ adrenergic receptor agonist methyldopa, acetylsalicylic acid, and levothyroxine but there was no significant difference between the two groups with respect to the usage of angiotensin-converting enzyme inhibitors, or angiotensin receptor blockers, Ca^2+^-channel blockers, imidazolidine or α_2_ adrenergic receptor agonists, statins, and diuretics. The possible modifying effect of β-blockers and methyldopa on the results of cardiovascular reflex tests is discussed in the limitations section.

### Laboratory data of infertile women with obesity and control subjects

#### Vitamin D

Unexpectedly, in our study, baseline plasma levels of 25OHD3/D2 vitamin were not significantly lower in female patients with obesity compared to the controls, although the trend clearly indicated lower 25OHD3/D2 vitamin levels in the infertile patient with obesity group (**Table 2**). Contrary to our findings, data from meta-analyses consistently demonstrate an inverse relationship between vitamin D levels and body weight (37, 38). According to the baseline laboratory test showing low 25OHD3/D2 vitamin levels, D3 vitamin loading and subsequent supplementation were initiated in 7 out of 16 patients with obesity, leading to a significant improvement in 25OHD3/D2 vitamin levels at the 2^nd^ measurement. In randomized trials (39), weight loss resulted in a greater increase in serum 25-hydroxyvitamin D levels compared to weight maintenance. In the randomized, placebo-controlled trial by Holt et al. (40), originally designed to evaluate the effectiveness of liraglutide, exercise, or their combination in weight loss maintenance among women, weight loss induced by a low-calorie diet led to an increase in serum 25(OH)D levels. In our study, due to the medical correction of low baseline vitamin D levels through oral supplementation, the specific impact of weight loss therapy on serum 25OHD3/D2 vitamin levels cannot be clearly determined. However, by the time of follow-up measurements, 25OHD3/D2 vitamin levels statistically significant increased compared to baseline.

#### Inflammatory markers

Multiple preclinical and clinical studies have confirmed that chronic low-grade inflammation in adipose tissue is linked to metabolic diseases and organ tissue complications in obese individuals (41). As observed in our research, the CRP value was significantly higher in patients with obesity compared to controls with a normal value of BMI. Several studies, including our own (5) have demonstrated a strong association between elevated CRP levels and obesity (42, 43), which is found to be more pronounced in women compared to men (42). Additionally, other inflammatory markers, such as white blood cell and platelet counts were found significantly higher in individuals with abnormal BMI, particularly among women (42). In our study, white blood cell, platelet, and red blood cell counts were all significantly higher in infertile patients with obesity compared to controls with a normal BMI value. In epidemiological studies, although obese individuals have higher white blood cell counts, these levels usually remain within the normal range, as evidenced by our research. Samocha-Bonet et al. (44) found that obese females had significantly higher platelet counts than normal-weight females, while no significant increase was observed in obese males. In our patients with obesity, the red blood cell count was significantly higher, while hemoglobin and hematocrit values were also elevated compared to the controls; however, these latter differences did not reach statistical significance. Nonetheless, the observed differences in red blood cell count are consistent with the literature (45). Our infertile patients with obesity exhibited significantly lower serum iron levels, elevated ferritin levels, and reduced mean cellular volume of red blood cells compared to the controls. This finding is consistent with the literature as obesity is associated with iron deficiency and elevated serum ferritin levels (46).

#### Dyslipidaemia, liver enzymes, carbohydrate metabolism, hyperuricemia

Obesity is linked to liver dysfunction through various mechanisms (47). In our study, serum levels of liver function markers such as ASAT/GOT, ALAT/GPT, GGT, and alkaline phosphatase (ALP) were significantly higher in female patients with obesity compared to the control group. Additionally, lipid abnormalities, as found in the present study, including elevated triglyceride and LDL-cholesterol levels, along with low HDL-cholesterol, are commonly observed in patients with obesity (48). Obesity is a recognized risk factor for developing carbohydrate metabolism disorders (49). Consequently, in our patient cohort, the prevalence of IGT and of type 2 diabetes as well as fasting serum blood glucose, HbA1c%, insulin level and insulin resistance characterizing HOMA-IR were significantly higher compared to the control group. Following weight loss therapy, a significant improvement in HbA1c% levels was observed at the 2^nd^ measurement among the finishers. In this study, patients with obesity exhibited significantly higher serum uric acid levels compared the controls, a finding consistent with the literature (50). Elevated uric acid levels are recognized as a strong predictor of underlying comorbidities such as obesity, hypertension, and diabetes, and have been linked to non-alcoholic steatohepatitis (51).

#### Sex hormone levels, thyroid function

Given that blood samples from patients with obesity were not consistently collected on days 3–5 of the menstrual cycle, an objective comparison of FSH, LH, and estradiol hormone levels was not possible due to their cycle sensitivity. No statistically significant differences were observed in thyroid function among the groups, confirming the effective treatment of hypothyroidism to achieve a medically appropriate euthyroid state in the patient group with obesity. Additionally, no differences were found in androgen, cortisol, and prolactin levels between the groups. However, the significantly reduced SHBG levels observed in obese individuals are not surprising and are consistent with the literature. This reduction was expected due to the presence of obesity (52) and is also not surprising given the significantly higher prevalence of PCOS (53).

### Cardiovascular autonomic function tests of infertile women with obesity and control subjects at baseline, and following weight loss therapy

Valsalva ratio was lower among female patients with infertility and obesity compared to controls, despite both groups having mean Valsalva ratios within the normal range (≥1.21). Furthermore, the 30/15 ratio was found to be significantly lower in comparison to controls with normal BMI. Both of these discrepancies indicate early parasympathetic dysfunction in infertile female patients with obesity, which may also reflect the influence of higher sympathetic nervous system activity. As a result of the discrepancies in the two tests, there was a significant difference in the autonomic score, which reflects the severity of cardiovascular autonomic neuropathy. A recent study conducted on individuals with obesity and normal glucose tolerance revealed that an increased waist-to-hip ratio (WHR), indicating visceral adiposity within this cohort, was associated with impaired regulation of cardiac autonomic function by both the parasympathetic and sympathetic nervous systems (54). In our own recently conducted study involving 72 obese, non-diabetic female patients, we also confirmed the presence of parasympathetic dysfunction compared to controls (5). In the present study, however, we found no correlation between WHR and any of the neuropathy assessment tests. Furthermore, the results of cardiovascular autonomic measurements showed no statistically significant difference before and after weight loss therapy.

### Peripheral sensory function in infertile female patients with obesity and control subjects at baseline, and following weight loss therapy

The 128-Hz Rydel-Seiffer graduated tuning fork test revealed an impaired sense of vibration symmetrically across all four limbs at the level of radius and halluces in infertile patients with obesity compared to controls. Our previous study was the first to demonstrate impaired vibrational sensing in women with obesity (5).

A significant difference was observed in the peripheral sensory function only of the median nerve at the tested frequency 2000 Hz using the Neurometer, indicating a deteriorated function in large myelinated sensory fibers. An increase in case numbers might further validate the deterioration in small myelinated and unmyelinated sensory fibers, as evidenced by the elevated CPT values at 250 and 5 Hz when compared to individuals with a normal BMI. Regarding the results of peripheral sensory measurements, there was no statistically significant difference before and after weight loss therapy.

### Correlations

The correlations observed between BMI and baseline body weight, and the results of both the tuning fork and cardiovascular reflex tests across the entire population underscore the significant role of increased body weight as a pathogenic factor in the context of neuropathy.

### Results at baseline (1^st^ measurement) and after weight loss therapy (2^nd^ measurement) in infertile women with obesity

Only 16 female patients with obesity participated in the follow-up second measurement, representing 27.6% of this highly motivated weight reduction cohort. In our study, the therapy duration for patients with obesity who completed the second measurement (finishers) averaged 232.9 ± 170.86 days (approximately 7.6 months), though the standard deviation was considerable. According to the literature, the retention rate for weight loss programs at 6 months (26 weeks) is 22% (55).

Participants in our study achieved a mean weight loss of 15.2 kg (−14.6% of initial body weight), with a BMI reduction of 5.6 kg/m² (**Table 5**), substantially exceeding the average reductions reported in the meta-analysis by Barboza et al. (56) (−3.35 kg and −1.45 kg/m², respectively). Additionally, we observed significant improvements in HbA1c levels, which were not confirmed in this meta-analysis (56). While direct comparison has its limitations due to differences in study populations, treatment protocols, and baseline characteristics (e.g., other average age and higher baseline weight in our cohort), our results demonstrate a marked efficacy of our complex weight loss therapy. Our study also revealed significant reductions in PBF% (47.0 ± 3.81 to 42.5 ± 6.50; n = 13; p < 0.05) and waist circumference (108.7 ± 20.05 cm to 94.0 ± 13.58 cm; p < 0.05). These results are consistent with those of Elkind-Hirsch et al. (57), who, in a randomized, placebo-controlled phase 3 trial assessing liraglutide 3.0 mg in women with obesity and polycystic ovary syndrome, reported similar improvements in waist circumference (111 ± 2.2 cm to 101 ± 2.0 cm; p = 0.011) and PBF% (47.6 ± 0.8% to 46.0 ± 0.9%; p = 0.028). Notably, their study used dual-energy X-ray absorptiometry to assess body composition, which may affect direct comparability. Recent evidence indicates that body composition parameters offer enhanced predictive value in evaluating reproductive potential (58, 59). Similar to the significant reductions in systolic and diastolic blood pressure observed in the studies by Barboza (56) and Astrup (60), a decreasing trend was also noted in our study, although it did not reach statistical significance. In contrast, in our study, cardiovascular autonomic and peripheral sensory function measurements showed no statistically significant differences before and after the weight loss therapy. To the best of our knowledge, no previous studies have investigated these functions specifically in infertile patients with obesity, based on currently available literature. With an increasing sample size, we anticipate that the alterations identified in our baseline cross-sectional analysis—namely, the significantly reduced Valsalva and 30/15 ratios compared to normal-BMI controls, the decreased peripheral vibration perception in all four limbs, and the elevated current perception threshold of large myelinated sensory fibers in the median nerve—will also improve in parallel with the overall metabolic enhancement achieved through weight loss therapy.

The pregnancy rates per oocyte retrieval in IVF treatments vary in Europe depending on the specific clinic, the age of the patients, and other medical factors. However, the general rates are well-documented in the scientific literature. According to reports from the ESHRE, the pregnancy rate per oocyte retrieval typically ranges between 20-30% (61).

In light of this, our own results appear favorable, though conclusions must be made cautiously due to the small sample size. Out of 16 patients, 3 became pregnant spontaneously, meaning IVF was not required for 18.75% of the cohort. Excluding these 3 patients, 5 out of the remaining 15 did not proceed with IVF therapy. Among the 8 patients who underwent IVF, 5 achieved clinical pregnancy representing a 62.5% success rate.

## Conclusion

Peripheral sensory neuronal impairments were detected in female patients with obesity compared to the controls with normal BMI. Cardiovascular autonomic dysfunction, indicated by the 30/15 and Valsalva-ratio, suggested the presence of parasympathetic dysfunction in these patients with obesity. Preconceptional weight loss in obese, infertile women significantly improved metabolic parameters. However, no statistically significant difference in cardiovascular autonomic or peripheral sensory function were observed before or after weight loss therapy. Among the infertile female patients who reached their preconceptional target weight, 18.75% achieved spontaneous pregnancy and did not require IVF therapy. Additionally, 62.5% of those who completed the program and underwent IVF therapy successfully conceived, exceeding the average success rate. Although the small sample size may limit the strength of our conclusions, the observed trends in our study are noteworthy.

## Limitations

We retrospectively analyzed the collected data, acknowledging that the program was a real-world intervention. It is imperative to take into account that significantly more patients with obesity took metformin, β-blockers, and α_2_ adrenergic receptor agonist compared to the controls. Beta blockade decreases sympathetic nerve activity, so it might attenuate the degree of abnormality in the achieved reflex test results in the patient group with obesity. In the case of metformin, we can also expect a similar masking effect through optimizing carbohydrate metabolism. Among the α_2_ receptor agonists, only methyldopa was used in the patients with obesity, which decreases sympathetic nervous system tone (62). Additionally, systemic administration of α-methyldopa in rats was shown to reduce mean arterial blood pressure, initially causing a short-term increase in heart rate followed by a prolonged decrease (63). Notably, these studies did not report an increased risk of orthostatic hypertension (64). Due to these effects, the use of metyldopa may mask sympathetic over activity in the obese patient group and, given its rare, but potential orthostatic hypotensive effect, may lead to poorer performance in sympathetic tests in the obese patient group.

The study initially included 58 patients, but only 16 patients completed the therapy. The difference in sample size may have a dual impact: on one hand, subtle but potentially meaningful differences may not reach statistical significance; on the other, small yet statistically significant changes may be subject to greater uncertainty. To account for the implications of limited sample size, we reported the statistical power associated with each significant finding. A power exceeding 80% supports the interpretation that these significant results are likely to reflect true underlying effects. Nevertheless, a greater sample size may be needed to increase the statistical power in several dimensions of the study and allow for a more comprehensive investigation of the relationship between the presence of obesity and other potential factors and the presence and the potential changes in neural function. Due to this limitation the investigation was extended, and a greater emphasis was put on the differences of obese infertile patients and normal weight controls with no known fertility problem. The low number of the patients finishing the therapy also impacted the investigation on fertility, due to the low sample size conclusive statistical analyses could not be performed on the medical data, and the results are mainly limited to fertility rates. Further research is warranted to investigate the effect of lifestyle interventions including nutrition coaching and physical training on neuropathy.

## Data Availability

The original contributions presented in the study are included in the article/**Supplementary Material**. Further inquiries can be directed to the corresponding author.
